# Superior Mesenteric Artery Syndrome: Delayed Diagnosis of a Rare Clinical Entity With a Common Clinical Presentation

**DOI:** 10.7759/cureus.26728

**Published:** 2022-07-11

**Authors:** Muhammad Hanif, Riaz Siddiqui, Ayesha Javed, Muhammad Ali, Omama Farooq, Mishal Fatima, Hajrah Farooq

**Affiliations:** 1 Surgical Unit II, Benazir Bhutto Hospital, Rawalpindi Medical University, Rawalpindi, PAK; 2 Surgery, Islamabad Medical Complex, Islamabad, PAK; 3 Internal Medicine, Islamabad Medical Complex, Islamabad, PAK; 4 General Surgery, Islamabad Medical Complex, Islamabad, PAK

**Keywords:** superior mesenteric artery syndrome, postprandial pain, intestinal obstruction, duodenojejunostomy, wilkie's syndrome

## Abstract

Superior mesenteric artery (SMA) syndrome, also known as Wilkie’s syndrome, is an uncommon disorder that involves a set of symptoms that primarily includes postprandial pain at times associated with intestinal obstruction. Although a rare disease in the general population, SMA syndrome has a high probability of occurrence in patients who are severely malnourished or have certain debilitating conditions leading to a loss of retroperitoneal fat. Here, we present the case of a 16-year-old male with a one-year history of postprandial abdominal pain associated with nausea, multiple episodes of vomiting, and abdominal distension. Amid a delayed diagnosis and multiple hospital visits, the patient’s condition further deteriorated. Thereafter, computed tomography of the abdomen confirmed this rare diagnosis. Because the patient could not be further managed conservatively, laparoscopic duodenojejunostomy was planned and done. This case report highlights the various challenges in diagnosing this disease and highlights the importance of an early diagnosis so that patients can be managed effectively and timely.

## Introduction

Superior mesenteric artery (SMA) syndrome, a very rare cause of intestinal obstruction, mainly occurs when a reduced angle between the abdominal aorta and SMA compresses the transverse (third) portion of the duodenum. Most patients present with a prolonged history of the aforementioned symptoms; however, it is not necessary that the cardinal symptoms of intestinal obstruction such as pain, vomiting, distention, and constipation be present in every patient. Therefore, both clinical and radiological evidence aid in the diagnosis [1].

## Case presentation

**Figure 1:**
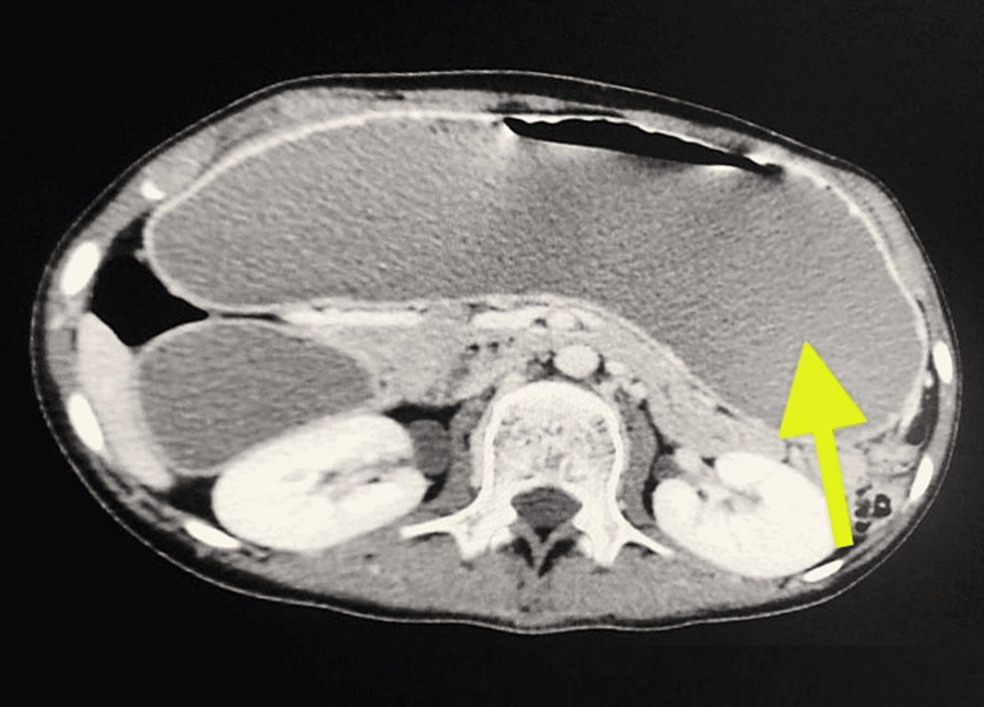
Computed tomography scan of the abdomen with oral contrast in the axial view showing gross dilatation of the stomach and retention of the oral contrast (yellow arrow).

**Figure 2:**
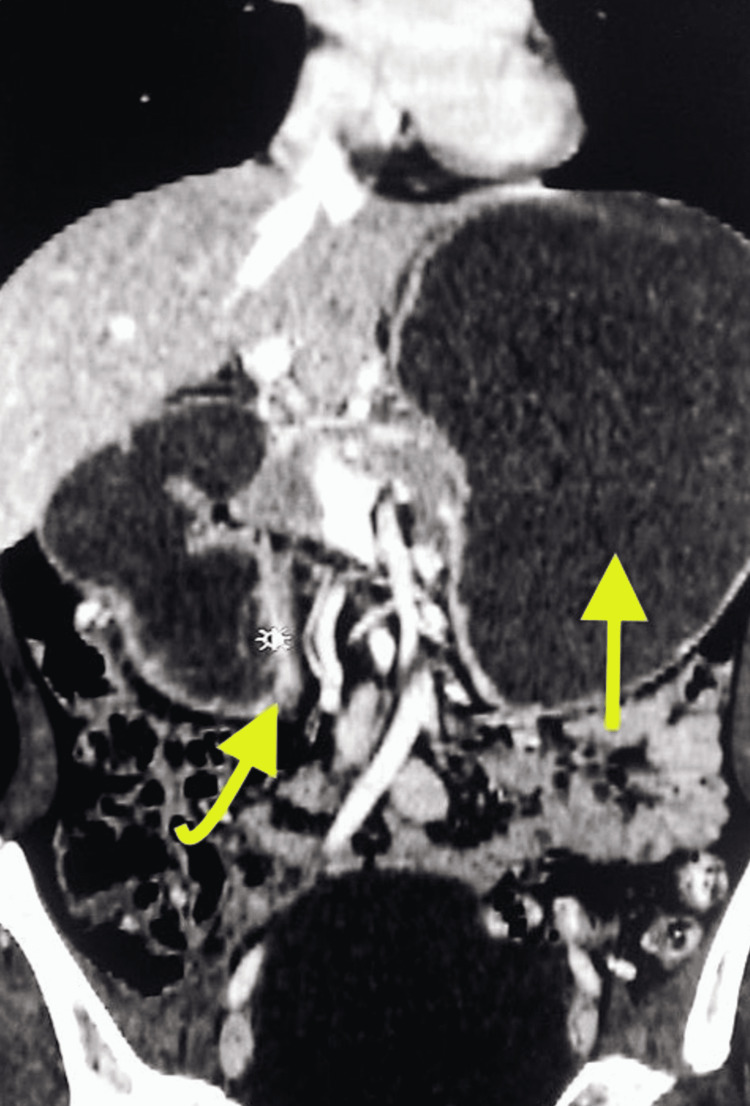
Computed tomography scan of the abdomen in coronal image showing dilated stomach (straight arrow) and dilated second portion of the duodenum (curved arrow with pointer).

**Figure 3:**
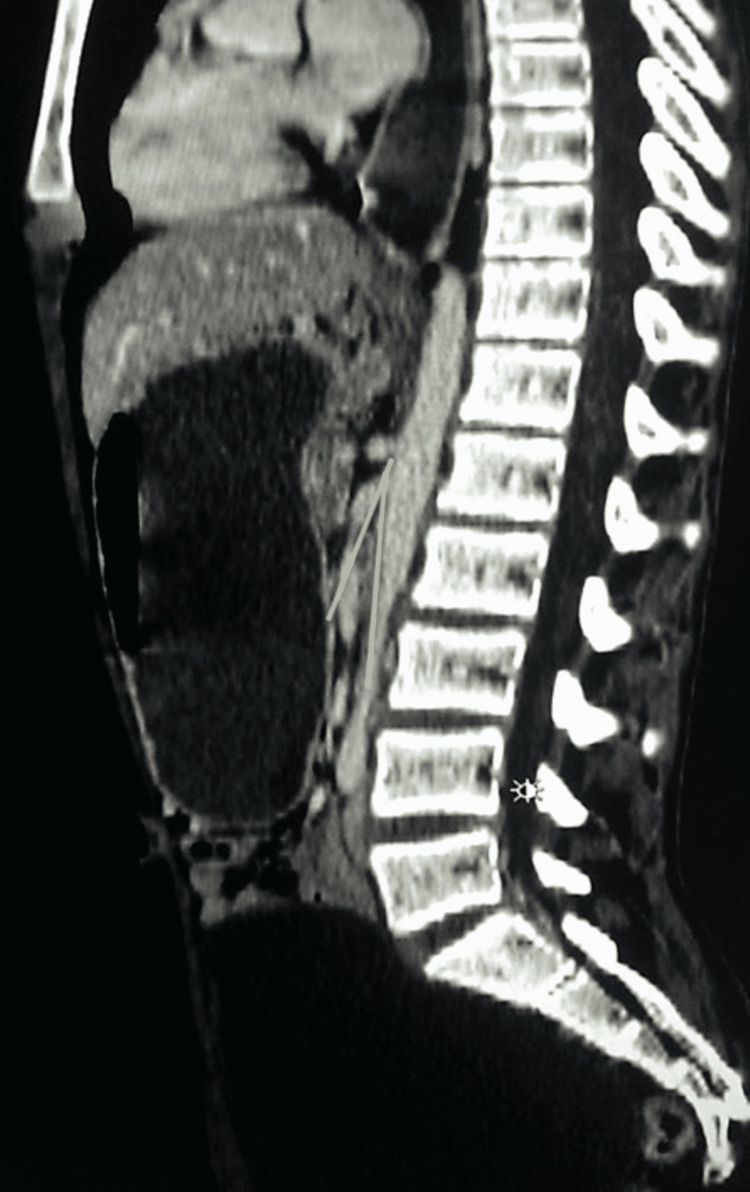
Computed tomography scan of the abdomen in sagittal view with two yellow angled lines showing the reduced aorto-mesenteric angle.

**Figure 4:**
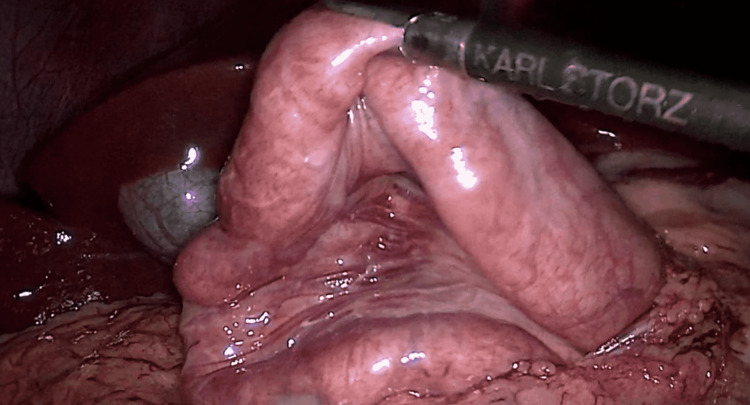
Laparoscopic duodenojejunostomy showing anastomoses being performed between the duodenum and jejunum, with the portion of the jejunum lifted.

A 16-year boy, with no known comorbidities, presented to the Medicine Outpatient Department with a history of postprandial abdominal pain for the past year. The pain was associated with nausea, multiple episodes of non-bilious, foul-smelling vomiting, and abdominal distension that became more frequent. He sought medical advice over the year. He had multiple outpatient and in-patient hospital visits but to no avail. The patient was treated conservatively with proton pump inhibitors and anti-emetics. His condition aggravated over the last three months with vomiting associated with meals and massive weight loss. On presentation to the hospital, he had an emaciated appearance with a body mass index (BMI) of 11 kg/m^2^. However, the abdominal examination was unremarkable. Biochemical and hematological profiles were within the normal range. Upper gastrointestinal endoscopy revealed gastroesophageal reflux disease, Los Angeles classification grade C. Computed tomography scan of the abdomen revealed gross dilatation of the distal esophagus, stomach, and proximal duodenum (Figures 1, 2), along with smooth tapering during the passage of the aorto-mesenteric interval (Figure 3) and collapsed distal duodenal and jejunal loops with a reduction in the aorto-mesenteric distance (4.5 mm) with borderline aorto-mesenteric angle (20 degrees) (Figure 3). The patient was shifted under the care of the surgical team. Central venous access was established and nutritional (nasogastric feed and parenteral) build-up was charted. After the pre-anesthesia assessment, he was prepared for laparoscopic duodenojejunostomy. He underwent the surgery (Figure 4) successfully and remained stable postoperatively. Oral intake was allowed on the fourth postoperative day, which the patient tolerated well. He was discharged home on oral medications eight days after the surgery.

## Discussion

The SMA is one of the major branches of the descending abdominal aorta arising anteriorly at the level between L1 and L2 intervertebral disc approximately 1 cm below the celiac trunk, perfusing the midgut from the second part of the duodenum to the proximal two-thirds of the transverse colon [2]. In SMA syndrome, the angle at which the SMA arises from the descending abdominal aorta is of high clinical significance. The normal angle between the aorta and SMA ranges from 25 to 60 degrees; meanwhile, different studies predict a mean angle ranging from about 20 to 70 degrees and an aortomesenteric distance ranging from about 10 to 28 mm. Reduction of this angle and aortomesenteric distance to about 6-16 degrees and 2-8 mm, respectively, results in the manifestation of characteristic signs and symptoms related to this syndrome [1].

As reported by previous studies, the prevalence of this syndrome is very low at 0.013-0.3%; in addition, these figures are believed to be understated [3,4]. Females aged between 10 and 39 years generally have a greater predisposition toward the SMA syndrome [5]. Symptoms normally comprise postprandial abdominal pain involving the epigastric region associated with early satiety, nausea, vomiting, bloating, burping, reflux, and abdominal distension. These symptoms are usually precipitated after meals which can lead to further weight loss [6].

The etiological factors involved can be mainly classified into three main categories such as congenital and surgical/orthopedics and factors influencing excessive weight loss. Congenital conditions include abnormalities in the position, angle, and distance of the SMA from the aorta [7]. Surgical causes include the precipitation of SMA syndrome in patients undergoing corrective surgery for scoliosis and those who underwent bariatric surgery and esophagectomy due to rapid weight loss [8]. The third category comprises ailments leading to severe weight loss such as malignancies, malabsorptive syndromes, anorexia nervosa, burns, drug abuse, human immunodeficiency virus/acquired immunodeficiency syndrome, and all other conditions promoting cachexia [9]. Nevertheless, SMA syndrome gets aggravated alongside malnourishment which substantially reduces the retroperitoneal fatty cushion support [10]. Like our case, patients with SMA syndrome may present chronically with a longstanding history of either persistent or intermittent gastrointestinal tract obstructive symptoms; hence, this syndrome is usually a diagnosis of exclusion, considered after extensive evaluation [9,11].

Various differential diagnoses to be kept in mind, particularly when dealing with adolescents or young individuals, include malrotation of the gut, para-duodenal hernias, bezoars, and Crohn’s disease [7]. Other differentials with a broader age group includes more common conditions such as pancreatitis, duodenitis and peptic ulcer disease, porphyria, and abdominal vessel aneurysm [4]. The general criteria (Hayne’s criteria) set to diagnose SMA syndrome are met when there is radiological evidence of proximal duodenal dilatation due to distal obstruction with an abrupt cutoff in the third portion of the duodenum. Additionally, the contrast medium flows in a backward direction due to distal duodenal obstruction accompanied by a delay in gastro-duodenojejunal emptying time and, lastly, the symptoms of intestinal obstruction getting better in the knee to chest or left lateral position [12].

Initial investigations may include upper gastrointestinal endoscopy which rules out ulcerative and inflammatory conditions. Characteristic findings of gastroscopy pointing toward SMA syndrome may show an increased diameter of the duodenum due to distal obstruction, accompanied by absent or reduced peristaltic waves with evidence of gastroesophageal reflux [4,10]. Ultrasonography can help elucidate narrowing of the aorto-mesenteric angle [4]. Contrast-enhanced studies (barium) might help identify the level of intestinal obstruction showing dilatation of the proximal part of the duodenum due to distal obstruction and cutoff of the contrast at the third part of the duodenum [13]. However, various other diseases such as scleroderma, pancreatitis, peptic ulcer, and malignant lymph nodes manifest similar radiologic findings, and therefore, are not used so frequently [14].

CT and angiography are widely used diagnostic investigation procedures for the assessment of the aorto-mesenteric angle and distance. An aorto-mesenteric angle of about 22-25 degrees and a distance of 8 mm on contrast-enhanced CT are findings suggestive of SMA syndrome [15].

Treatment options include both medical and surgical interventions [9]. Medical treatment is attempted first where a trial of conservative management is recommended. This revolves around passing a nasogastric tube to help attain gastric and duodenal decompression, resuscitation through intravenous fluids, and monitoring and replacing serum electrolytes. Meanwhile, the main goal of a physician is to resolve the underlying conditions aggravating SMA syndrome and weight gain. Weight gain can be achieved through total parenteral nutrition, and prokinetics may be beneficial in improving weight gain [15,16]. For patients who have a short duration of history, conservative treatment works best to resolve the signs and symptoms of SMA syndrome; however, when medical treatment fails, surgical management is usually required [16].

Surgical options include gastrojejunostomy, duodenojejunostomy, and Strong’s procedure. The current and most common surgical method adopted is laparoscopic duodenojejunostomy, which includes anastomosis between the second portion of the duodenum and proximal jejunum, thus establishing an effective decompression of the duodenum. This procedure carries with it the benefit of rapid recovery, functional improvement in bowel motility and patient’s health, decreased chances of small bowel adhesions and postoperative incisional hernia, minimum blood loss, less pain postoperatively, and, lastly, a good cosmetic outcome [17].

## Conclusions

Although being a very rare cause of intestinal obstruction, this diagnosis should not be excluded, especially in patients who have the above-described symptoms and risk factors. A delay in diagnosis can not only affect the patient’s mental health but also the willingness to cope with adverse effects. The diagnostic workup mainly includes CT and angiography. Patients can be managed conservatively when diagnosed earlier but surgical intervention is necessary for a delayed diagnosis or when medical treatment fails.
