# Correction: Superior Mesenteric Artery Syndrome: Delayed Diagnosis of a Rare Clinical Entity With a Common Clinical Presentation

**DOI:** 10.7759/cureus.c73

**Published:** 2022-08-29

**Authors:** Muhammad Hanif, Riaz Siddiqui, Ayesha Javed, Muhammad Ali, Omama Farooq, Mishal Fatima, Hajrah Farooq

**Affiliations:** 1 Surgical Unit II, Benazir Bhutto Hospital, Rawalpindi Medical University, Rawalpindi, PAK; 2 Surgery, Islamabad Medical Complex, Islamabad, PAK; 3 Internal Medicine, Islamabad Medical Complex, Islamabad, PAK; 4 General Surgery, Islamabad Medical Complex, Islamabad, PAK

This article was submitted and published without the knowledge of the senior faculty directly involved in the management of the case. An internal investigation was conducted by the Islamabad Medical Complex Department of Surgery and it was determined that although the submitting authors did neglect to notify and include the appropriate authors, this was done out of ignorance and did not constitute intentional academic fraud. At the request of the senior faculty involved in the case, they have been added as the first, second and third authors. The published article has been updated to include the following author list:

1. Muhammad Hanif (Corresponding Author) Surgical Unit II, Benazir Bhutto Hospital, Rawalpindi Medical University, Rawalpindi, PAK
2. Riaz Siddiqui Surgery, Islamabad Medical Complex, Islamabad, PAK
3. Ayesha Javed Surgery, Islamabad Medical Complex, Islamabad, PAK
4. Muhammad Ali Internal Medicine, Islamabad Medical Complex, Islamabad, PAK
5. Omama Farooq Internal Medicine, Islamabad Medical Complex, Islamabad, PAK
6. Mishal Fatima General Surgery, Islamabad Medical Complex, Islamabad, PAK
7. Hajrah Farooq Internal Medicine, Islamabad Medical Complex, Islamabad, PAK

